# Supplementary material for: Experimental demonstration of high space compression by optical spaceplates
Source: Nat Commun. 2026 Apr 20;17:3493. doi: 10.1038/s41467-026-71500-1 (PMC13096489; doi:10.1038/s41467-026-71500-1)
Supplement: Supplementary file 1 — Supplementary Information [file 41467_2026_71500_MOESM1_ESM.pdf]

Supplementary Method 1: Design and Characterization

We now discuss how we determine the compression factor $\mathcal{R}$ from simulations of each of the devices found from gradient-descent (GD) design, and Fabry-Perot cavity (FPC) design methods. The lateral shift w is derived from the device transmission phase, ϕ_t, and establishes a relationship with the angular dependence of the device:

$$w = -\frac{\partial \phi_t}{\partial k_x} = \frac{-1}{k\cos(\theta)}\left(\frac{\partial \phi_t}{\partial \theta}\right)_{\theta_0}, \quad (1)$$

where θ is the incident angle. For a plate made of a uniform optical material, the lateral shift w scales with $\tan(\theta)$. Considering a plate with refractive index $n = 1$ (i.e., free space), and thickness d, the lateral shift would be $w = -d\tan\theta \equiv -w_a$. An ideal spaceplate will thus induce a lateral shift of $w = -d_{\text{eff}}\tan\theta = -\mathcal{R}d\tan\theta$. Whereas the lateral shift is measured along the exit interface, the transverse shift Δx is measured as the distance perpendicular to the beam or ray. Rather than tilting the incident beam, we leave the beam stationary along z and instead tilt the device so that its surface normal is an angle θ to the incident beam. This leads to an additional lateral shift w_a that is purely geometric due to the frame change. The total lateral shift is thus $w + w_a$. The transverse shift is the projection of this total lateral shift onto the x direction,

$$\Delta x = (w + w_a)\cos\theta, \quad (2)$$

which is valid for both the ideal spaceplate lateral shift and the lateral shift predicted from a device design, Eq. 1. In the ideal case, we arrive at Eq. 1 of the main text, $\Delta x = -(\mathcal{R}-1)d\sin\theta$. For small angles, the linear negative slope of Δx vs θ is proportional to $\mathcal{R}-1$. Determining this slope is our main experimental method to measure the compression factor.

The transmission phase of the device can be determined using the transfer-matrix method (TMM) to analyze the multilayer stacks' properties as a whole, accounting for their layer thicknesses and optical properties such as the refractive index and absorption. Using TMM, the transmittance and transmission phase can be determined for a device as a function of incident angle or optical wavelength λ.

An ideal spaceplate transmission phase ϕ_t is given by

$$\phi_{\text{SP}} = \frac{2\pi n_{\text{BG}}}{\lambda}\mathcal{R}d\cos(\theta), \quad (3)$$

where $n_{\text{BG}} = n_{\text{air}} = 1$ is the background index, $d_{\text{eff}} = \mathcal{R}d$ is the effective thickness of the device, and λ is the input wavelength. By comparing to this ideal phase, we can determine the spectral response of the compression factor, as well as the device numerical aperture. The summary of device characterization from TMM simulation is shown in Figure S1.

Over a small angle range, a device's transmission phase should be quadratic in angle to match an ideal spaceplate. The range of angles where the phase remains quadratic sets the device's working-angle range. Over this range, $\mathcal{R}$ is extracted from a fit to the ideal spaceplate phase. The device in the inset of Fig. S1**c.** has four unit cells. All details of the devices and their design parameters can be found in Table I. The right axis of Fig. S1**a.** shows the simulated phase, which fits quadratically to the ideal spaceplate phase in the highlighted blue region, and consequently, sets the operating angular range of the device. The left axis of Fig. S1**a.** shows the corresponding angular dependence of the transmittance. Figure S1**b.** shows the angular dependence of the transmission phase for the simulated TMM response, and its corresponding fit using an ideal spaceplate phase. The calculated lateral walk-off is shown in Fig. S1**c.**, following Eq. (1) and substituting the results from Fig. S1**b.** The wavelength dependence on compression ratio (left) and transmittance (right) are shown in Fig. S1**d.** For each wavelength, λ, $w(\theta)$ is calculated and fit using Eq. (1) of the main text corresponding to the spaceplate walk-off from which $\mathcal{R}(\lambda)$ is extracted over the region of negative slope, highlighted in blue. Note that transmittance peaks correspond to peaks in the compression factor, indicating the resonance-type behavior enhancement of the compression factor.

All the devices have been fabricated on a 3-mm-thick

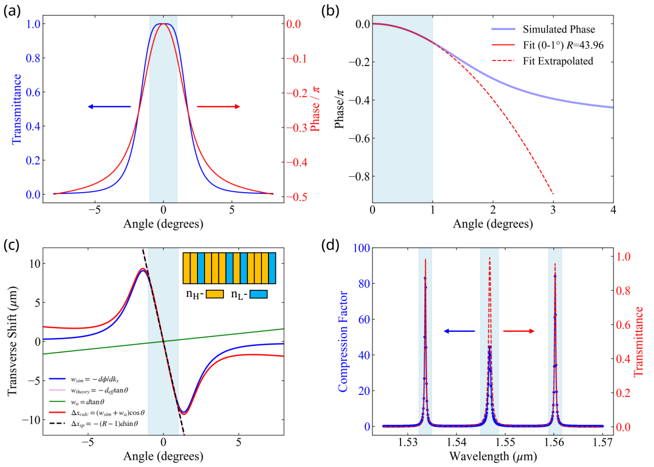

FIG. S1. Simulation of the FPC2 spaceplate. Using the TMM method, we calculate and plot the following spaceplate characteristics. **a.** Angular dependence of the transmittance (blue) and the transmission phase (red). **b.** Transmission phase (blue) and a fit to an ideal spaceplate phase (red) (See. Eq. 3) over the numerical aperture of the device highlighted in light blue. **c.** Calculated lateral shift (for small angles, this is approximately equal to the transverse walk-off) using Eq. 1 and schematic of the unit cell of the FPC2 spaceplate design, as seen in the main text. **d.** Transmittance spectrum (red) showing three resonance peaks, overlaid with corresponding spectral dependence of the compression factor (blue), $\mathcal{R}$. Using the fitting method in b for a given wavelength, the $\mathcal{R}(\lambda)$ can be extracted. The compression factor exhibits similar bandwidth and resonance positions to the transmittance peaks.

Device	Unit Cell Configuration	Unit Cells	Device Length (μm)	Effective Length (μm)	Compression Factor	Numerical Aperture (°)
FPC1	[2L, H, 3L, H]	8	13.10	43.76	3.368	3.5
FPC2	[2L, H, 3L, H, L, H, 3L, H]	4	12.04	767	43.0	1.0
GD1	Gradient Descent	17*	3.55	44.7	18.0	10.0
GD2	Gradient Descent	49*	14.48	3196	238.2	1.0

TABLE I. **Summary of device parameters, performance, and configuration.** FPC1 and FPC2 comprise integer-valued multiples of quarter-waves for low and high index layers represented as L and H, respectively, and two other devices of an alternating index, with thicknesses determined by gradient descent. The number of unit cells indicates the repetition count of the unit cell. The compression factor denotes the ratio of the effective to real device length. The number of layers is indicated with a star for the gradient descent devices (See Table II for more details). The device's angular range is also displayed. Note that all devices are quoted for p-polarized light, although s-polarized light exhibits comparable performance in a limited angular range of $\theta_{\text{device}} \leq 10°$.

substrate to simplify the isolation of the spaceplate effect. A bare substrate can deviate the light as a result of the inherent Transmitted Wavefront Error (TWE). In addition, at a non-zero angle of incidence, the offset of the beam will depend on substrate thickness. To minimize these effects, the substrates were fabricated to an etalon quality, which in modern systems can reach $< \lambda/100$ in surface flatness and parallelism of the two surfaces.

Thinner substrates could have been used for this proof-of-concept study but it would have added complexity that is extraneous to action of the spaceplates we wish to characterize. For example, thinner substrates are more susceptible to bending due to the stress created by the multilayer coating. To this point, all of the fabricated devices had an AR coating applied to surface of the substrate that is opposite to the spaceplate in order to balance this stress. This is a standard procedure in commercial devices. This stress-balance coating reduces the substrate curvature and minimizes the reflection off the back surface of the device.

To distinguish between the effect of surface curvature and the spaceplate effect itself, we estimate the curvature that would cause a focal shift Δf that matches the observed shift Δ=0.5 mm that we attribute to the spaceplate. In the experiment, the spaceplate is placed after a focusing lens with focal length $f = 100$ mm. We model the substrate surface curvature r as a thin lens with focal length f'. This thin lens will shift the focus by the largest amount if the thin lens is placed adjacent to the focusing lens. The total focal length f_T of the two-lens system can be found using:

$$\frac{1}{f_T} = \frac{1}{f} + \frac{1}{f'}. \tag{4}$$

Consequently, the focal shift due to the addition of the thin lens is $\Delta f = f_T - f$. Using the parameter values given above, in order for $\Delta f = \Delta$, the focal length of the curved surface would need to be $f' \simeq 20$ m. From this focal length, we find the curvature r from the lens maker's equation:

$$\frac{1}{f'} = (n-1)\frac{1}{r}. \tag{5}$$

Using $n = 1.5$ for glass, $r \simeq 10$ m. Across the substrate aperture of 2.5 cm, this curvature would result in a change in surface height of 8 μm or 5λ for our devices, which is much larger than the substrate specification of $\lambda/100$. This estimate is a worst-case scenario. In the experiment, the spaceplate is roughly 100 mm from the focusing lens, and a curvature $r \simeq 10$ m would cause a much smaller focal shift than Δ=0.5 mm. In summary, the observed effect of the shift of the beam waist cannot be attributed to the lensing effects caused by the unintended curvature of the device.

Supplementary Method 2: Theoretical limits calculations

According to Shastri et al. (2022), there is a fundamental relationship between the bandwidth of the device $\Delta\omega$, its numerical aperture, NA, and compression ratio $\mathcal{R}$:

$$\frac{\Delta\omega}{\omega_c} \leq \frac{\eta_{max}}{2\sqrt{3}} \frac{\frac{v_{gx}}{c}}{\mathcal{R}\frac{NA}{n_b} - \frac{v_{gx}}{c}}, \tag{6}$$

where n_b is the refractive index of the background medium, c is the speed of light in the background medium, $v_{gx} = c \cdot \sin\alpha_m$ in a realistic limit, and $NA = n_b \sin\alpha_m$ (α_m - maximum acceptance angle) and $\eta_{max} = |\frac{\varepsilon_{max} - \varepsilon_{min}}{\varepsilon_b}|$ (η_{max} and η_{min} are the maximum and minimum permittivity at any point within the structure and ε_b is the permittivity of the background). Considering all the definitions, we can simplify the formula:

$$\frac{\Delta\omega}{\omega_c} \leq \frac{\eta_{max}}{2\sqrt{3}} \frac{1}{\mathcal{R} - 1}. \tag{7}$$

Using this inequality, we can estimate the limits on $\mathcal{R}$:

$$\mathcal{R} \leq \frac{\omega_c}{\Delta\omega} \frac{\eta_{max}}{2\sqrt{3}} + 1. \tag{8}$$

Theoretical limits on the compression factor of all devices are listed in Table I of the manuscript. We found that, under our conditions, the calculated limits are orders of magnitude lower than the theoretical limits for the fabricated device, which suggests that further design improvements are possible.

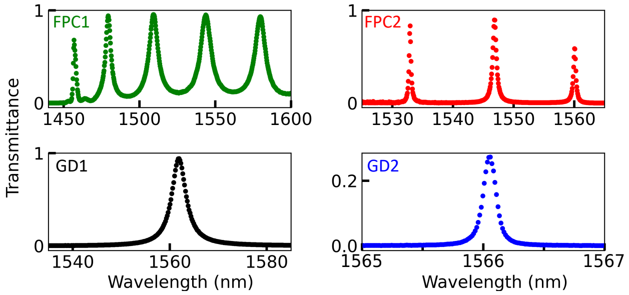

FIG. S2. **Measured transmittance of four devices.** FPC1 and FPC2 show side peaks due to multiple resonances based on integer-valued multiples of $\lambda/4$ layer thickness. Devices GD1 and GD2 show a single resonance peak due to layer thickness based on gradient descent. Designs were simulated using the TMM centred at $\lambda_{\text{device}} = 1550$ nm. Actual wavelengths differ from design wavelength due to fabrication tolerances, therefore $\lambda_{m,\text{FPC1}} \approx 1547$ nm, $\lambda_{m,\text{FPC2}} \approx 1531$ nm, $\lambda_{m,\text{GD1}} \approx 1562$ nm, and $\lambda_{m,\text{GD2}} \approx 1566$ nm. Devices were fabricated on top of 3 mm thick fused silica, with an anti-reflective coating on the films to minimize stress and maximize transmission. The lowest transmittance was measured to be approximately 25% at the peak of device GD2. Transmittance peaks correspond to regions of spatial compression, where the magnitude of spatial compression governs the device's resonance bandwidth and angular range.

Supplementary Method 3: Measured Device Transmittance

High transmittance is essential for most optical elements used in imaging applications. Figure S1(**c**) illustrates the transmittance versus input angle for FPC2, showcasing its high transmittance in the highlighted region for a given wavelength. The wavelength chosen is important since it is a resonant effect. While all devices were initially designed to work at $\lambda_{device} = 1550$ nm, fabrication intolerances shifted the central wavelengths to $\lambda_{m,\text{FPC1}} \approx 1547$ nm, $\lambda_{m,\text{FPC2}} \approx 1531$ nm, $\lambda_{m,\text{GD1}} \approx 1562$ nm, and $\lambda_{m,\text{GD2}} \approx 1567$ nm. The measured transmittance of each device is shown in Fig. S1. The transmittance of FPC1 is shown in green in Fig. S1, containing five peaks. However, we note there are seven peaks due to the criterion of $n-1$ peaks, where n is the number of unit cells for the FPC designs. Peaks were measured up to 1600 nm, highlighting the central peak, where measurements in the main text are taken. FPC2, GD1, and GD2 are shown in red, black, and blue, respectively. The drop in transmittance for GD2 is likely due to the large number of layers and fabrication intolerances compounding to an overall lower transmittance. From the measured transmittance, we deduce the appropriate range of wavelengths to measure for the compression factor.

Supplementary Method 4: Spaceplate as a filter in momentum space

The spaceplate has a narrow window of transmittance around normal incidence that can reduce the angular width of the transmitted beam. In turn, this will increase the waist of the transmitted beam, an effect we observe. Consider a beam with a transverse Gaussian field profile $g(x)$ that is incident on the spaceplate,

$$g(x) = g_0 \exp\left(-\frac{x^2}{w_0^2}\right), \tag{9}$$

where w_0 corresponds to the beam waist and $\tilde{g}_0$ is the normalization constant. The Fourier transform gives the same beam in terms of its transverse momentum distribution,

$$|\tilde{g}(k_x)| = \tilde{g}_0 \exp\left(-\frac{k_x^2}{\sigma_0^2}\right). \tag{10}$$

The spaceplate's transmittance $T(k_x) = |\tilde{t}(k_x)|$, where $\tilde{t}$ is the transmission amplitude. We approximate the action of the multilayer stack spaceplate as a Gaussian filter

$$|\tilde{t}(k_x)| = \tilde{t}_0 \exp\left(-\frac{k_x^2}{\sigma_T^2}\right) \tag{11}$$

where σ_T characterizes the momentum-space bandwidth of the filter and $\tilde{t}_0$ is the corresponding normalization constant. For both the spaceplate and beam functions, one can find a waist from a transverse momentum width, $\sigma = 2/\omega$. The field transmitted through the spaceplate will be,

$$|\tilde{h}(k_x)| = \tilde{g}(k_x)|\tilde{t}(k_x)| = \tilde{g}_0\tilde{t}_0 \exp\left(-\frac{k_x^2}{\sigma_h^2}\right), \tag{12}$$

where the transmitted beam with have a momentum width of

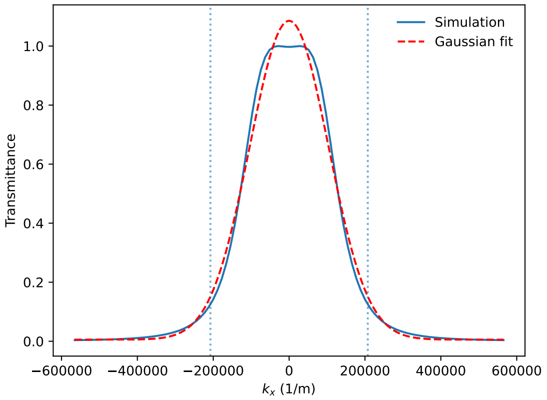

FIG. S3. **Spaceplate as a filter in momentum space.** The transmittance of the FPC2 device as a function of the transverse component of the wave vector (blue) k_x and a Gaussian fit (red) to determine the filter field half-width σ_T (blue dotted lines show the equivalent intensity full-width, $2\sqrt(2)\sigma_T$)

$$\sigma_h = \frac{\sigma_0 \sigma_T}{\sqrt{\sigma_0^2 + \sigma_T^2}}. \tag{13}$$

Using σ_h we find that the transmitted beam waist ω_h has increases by a factor of F over the incident the incident beam waist, where

$$F = \frac{\omega_h}{\omega_0} = \sqrt{\left(\frac{\sigma_T}{\sigma_0}\right)^2 + 1}. \tag{14}$$

We now estimate F and compare to experiment in our characterization of spaceplate device FPC2. The transverse momentum field width $\sigma_T = 2.1 \times 10^5$ m^{-1} (2.97°) is extracted from a Gaussian fit to the simulated transmittance as a function of k_x, as shown in Fig. S3. Our input beam is measured to have a waist of $\omega_0 = 38$ μm, which corresponds to a momentum width of $\sigma_0 = 0.53 \times 10^5$ m^{-1} (0.74°). With these values, we estimate that $F = 1.03$. For the experimental measurement of focal advance using FPC2, we observe that $w_h = 40$ μm, which results in an experimental factor of$F_{\text{exp}} = 1.05$. The two values are in good qualitative agreement, which suggests that the spaceplate's narrow transmittance window in transverse momentum transmission is the predominant cause of our observed increase in the transmitted beam waist.

Supplementary Method 5: Measured Lateral Shift for each FPC2 peak

The FPC spaceplate design shows multiple peaks in transmittance. Through TMM simulations, it was found that the compression factor also peaks at these resonance positions. As seen in Fig. S2, the peak transmittance values of the outer peaks are unequal, indicating a slight difference in the resonance. TMM simulations however, indicate symmetric transmittance about the central resonance. This is due to neglecting the small dispersion of the constituent materials on the wavelength range of interest. Therefore, the dispersion in turn affects the performance of the compression factor as well, showing different slopes for the lateral shift, and consequently $\mathcal{R}$, as in Fig. S2 for the green and blue curves, corresponding to left and right peaks, respectively. As for the red curve, the compression factor is lower than the outer peaks, and the slope is approximately half of the green curve, leading to a compression factor approximately half of the outer peaks.

Supplementary Method 6: Fabrication Details and Analysis

Devices were all designed to thicknesses of approximately 12 μm or less, except GD1, which was about four times smaller. Devices were grown on fused silica of thicknesses $d = 3.03$ mm for FPC1, FPC2, and GD2, and $d = 2.95$ mm for GD1. Alternating high and low indices comprise silicon ($n_H = 3.2$ at 1550 nm) and silica ($n_L = 1.456$ at 1550 nm), respectively. The films comprising the multilayer stack were deposited using magnetron sputtering. An anti-reflective coating was placed on the device to minimize reflection and counteract any curvature of the sample due to the stress of the films after growth. Device performance was calculated by TMM using design parameters summarized in Table I. Layer thicknesses, materials, and refractive indices for the design of GD1 and GD2 are given in Table II.

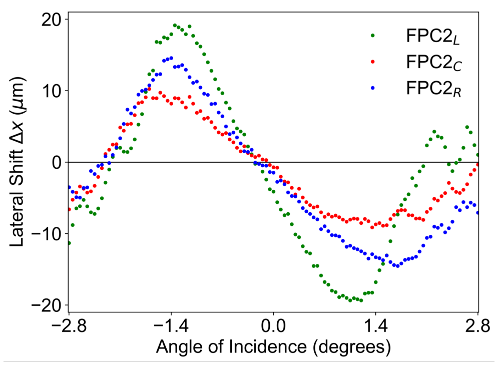

FIG. S4. **Measured lateral shift at FPC2 peak positions.** The lateral shift is measured over two times the numerical aperture of FPC2 at the peak position for each resonance seen in the transmittance spectrum, (See Fig. S2, red curve). The red curve (FPC2$_C$) indicates the lateral shift for the central peak, with a slope less step than the outer peaks. Left and right peaks are plotted in green (FPC2$_L$) and blue (FPC2$_R$) respectively.

Supplementary Method 7: Polarization response of the devices

Figure S5 shows the polarization response for all devices, extracted using TMM. Maximum compression ratio discrepancy for the low-NA devices (GD2, FPC1) reaches 10 %, while devices with higher NA (GD1, FPC2) reach 40%.

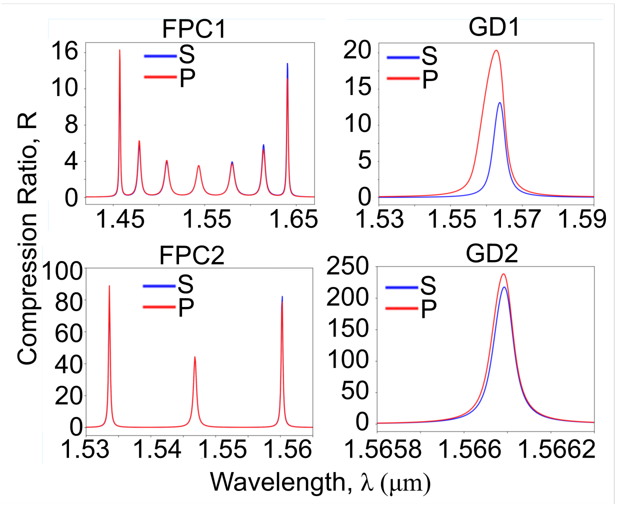

FIG. S5. **Simulated polarization response.** Compression factor is calculated for all the devices over the transmission range with s- and p-polarized input light.

TABLE II. **GD1 and GD2 design.** Layer thicknesses from the gradient-descent optimization. Odd layers are a-Si:H ($n = 3.196$) and even layers are SiO_2 ($n = 1.458$).

GD1		GD2			
Layer	d (μm)	Layer	d (μm)	Layer	d (μm)
1	0.1189	1	0.1227	26	0.2792
2	0.1959	2	0.2668	27	0.3490
3	0.1355	3	0.3872	28	0.1442
4	0.1942	4	0.3844	29	0.2740
5	0.1313	5	0.1438	30	0.4035
6	0.1912	6	0.2343	31	0.2060
7	0.1159	7	0.2940	32	0.2497
8	0.1107	8	0.2958	33	0.3440
9	0.0961	9	0.2250	34	0.2783
10	0.1278	10	0.1530	35	0.2660
11	0.1233	11	0.2290	36	0.3810
12	0.1753	12	0.2034	37	0.1640
13	0.1339	13	0.3430	38	0.3076
14	0.1880	14	0.4079	39	0.1610
15	0.1370	15	0.3720	40	0.1805
16	0.1897	16	0.2599	41	0.2080
17	0.1186	17	0.2050	42	0.3898
		18	0.2748	43	0.2985
		19	0.3353	44	0.1962
		20	0.3690	45	0.2233
		21	0.3170	46	0.3029
		22	0.2705	47	0.2800
		23	0.2860	48	0.2044
		24	0.3362	49	0.3535
		25	0.2580		

Supplementary Method 8: Angular Dispersion Analysis We performed numerical analysis of the angular dependence of the focal shift to explicitly show the link between Figs. 3 and 4 of the manuscript. Here we calculate the longitudinal shift directly from the angular dispersion data. 'Spaceplate + Glass' and 'Glass Only' traces correspondingly show negative longitudinal shifts (spatial compression) contributed by the devices GD1 and GD2, for contrast, that oppose the positive shift of the glass substrate. This explicitly confirms that the walk-off observed in Fig. 4 directly translates to the imaging results in Fig. 3, demonstrating that the device is indeed producing the specific angular dispersion required to shorten the imaging system

In devices GD1 and GD2, the angular dispersion generates a more constrained numerical aperture for a uniform focal shift, as the focal shift becomes non-uniform above a threshold of $\theta_{device}/2$ for GD2, and $\theta_{device}/4$ for GD1.

For FPC2 however, the focal shift is uniform over approximately the full device angular range, Since in the experiment we explicitly operated within the beam divergence limits where the device phase response is parabolic, the results are consistent with a uniform focal shift.

The longitudinal focal shift Δ is strictly the angular gradient of the transverse walk-off, $\Delta \sim \left(\frac{\partial w}{\partial \theta}\right)_{\theta_0}$. The angular derivatives of the transmission phase give the walk-off and focal shift effects, which serve as excellent, directly measurable methods for spaceplate characterization (i.e. negative-sloped transverse walk-off and retraction of the image plane near normal incidence).

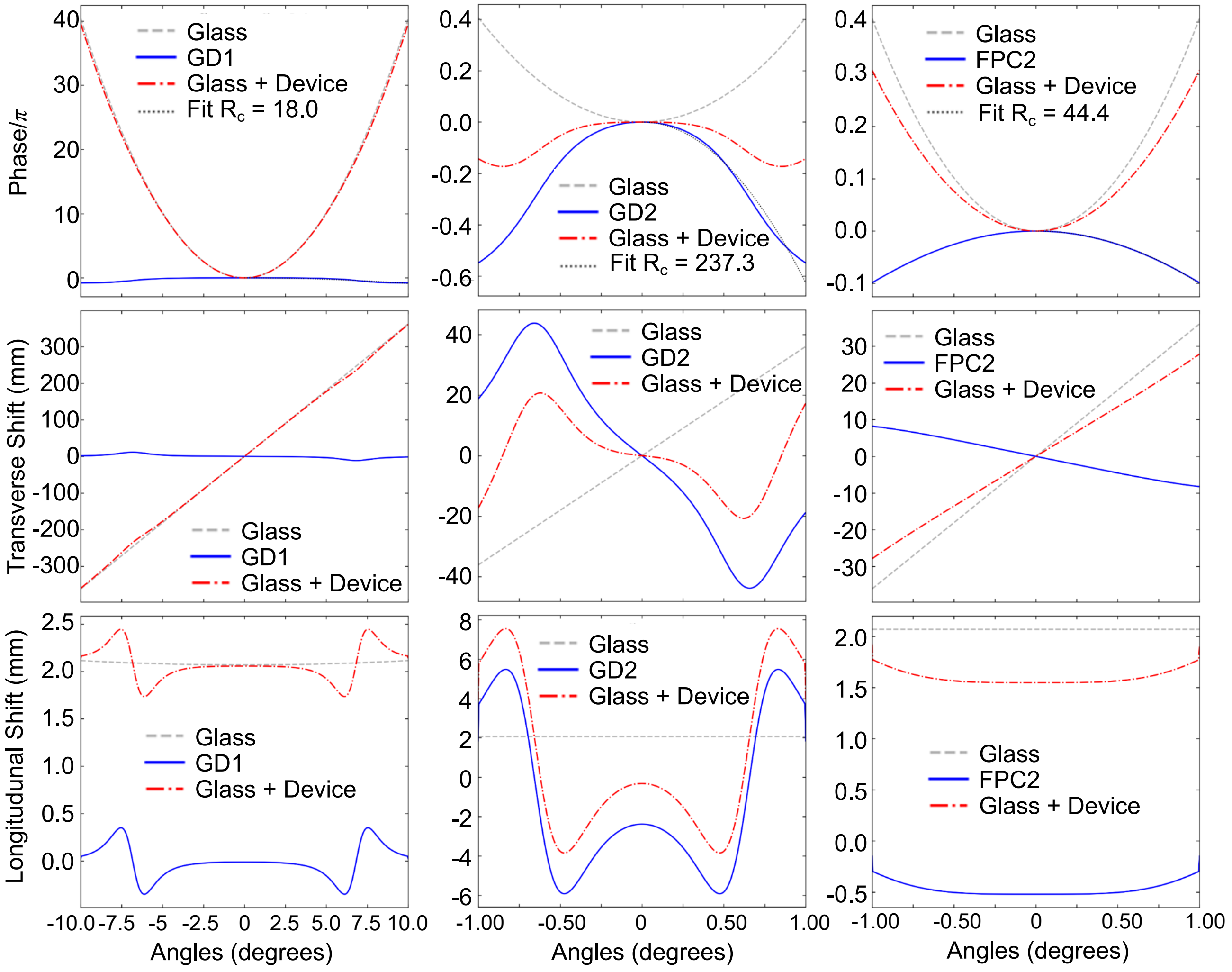

FIG. S6. **Angular dispersion analysis.** The angular dependence of Phase \ π, Transverse Shift and the Longitudinal Shift for Glass + Device (red), Glass (grey) and the device (blue, left to right column: GD1, GD2, FPC2)
